# Predicting the Evolutionary Variability of the Influenza A Virus

**Published:** 2017

**Authors:** T.A. Timofeeva, M.N. Asatryan, A.D. Altstein, B.S. Narodisky, A.L. Gintsburg, N.V. Kaverin

**Affiliations:** Federal State Budgetary Institution «N.F. Gamaleya FRCEM» of the Ministry of Health of the Russian Federation, Gamaleya Str. 18, Moscow, 123098, Russia

**Keywords:** Influenza A virus, phylogenetic trees, escape mutants, computational tools, computational modeling, phenotypic characteristics, reverse genetics

## Abstract

The influenza A virus remains one of the most common and dangerous human health concerns due to its rapid evolutionary dynamics. Since the evolutionary changes of influenza A viruses can be traced in real time, the last decade has seen a surge in research on influenza A viruses due to an increase in experimental data (selection of escape mutants followed by examination of their phenotypic characteristics and generation of viruses with desired mutations using reverse genetics). Moreover, the advances in our understanding are also attributable to the development of new computational methods based on a phylogenetic analysis of influenza virus strains and mathematical (integro-differential equations, statistical methods, probability-theory-based methods) and simulation modeling. Continuously evolving highly pathogenic influenza A viruses are a serious health concern which necessitates a coupling of theoretical and experimental approaches to predict the evolutionary trends of the influenza A virus, with a focus on the H5 subtype.

## A BIG PROBLEM FROM A SIMPLE THING CALLED “FLU”

The isolation of the human influenza virus was first reported by W. Smith, C.H. Andrewes, P.P. Laidlaw from the National Institute for Medical Research in England in 1933 [1, 2]. Two years before their report, in 1931 Richard E. Shope from the USA isolated a swine influenza virus [3, 4]. A considerable body of data regarding the structural and functional properties of influenza viruses, disease pathogenesis, adaptive and innate immune responses has been accumulated over the past 85 years. The human influenza virus has emerged as one of the primary public health threats due to its wide incidence and ability to cause a severe respiratory illness. Human influenza can lead to epidemics and pandemics, accompanied by high mortality rates and significant economic losses, because the influenza A virus exhibits rapid evolutionary dynamics and fast adaptation to human hosts that possess a general, non-specific immune system and vary in the levels of acquired immunity. Human influenza virus strains carry specific phenotypic characteristics that affect the disease process: i) the ability to attach to and infect the epithelium of the upper airway passages (receptor-binding activity), ii) the ability to escape the immune response, and iii) the ability to produce infectious virus progeny. The former two properties are mainly a factor of viral surface proteins, whereas input to the latter characteristic comes from the entire viral proteins. The virus undergoes phenotypic changes arising from genetic changes.

Following an infection, the virus particles are exposed to two types of immune response. The humoral immunity, mediated by neutralizing antibodies to the surface proteins hemagglutinin (HA) and neuraminidase (NA), plays an essential role in the host defense. Anti-HA antibodies bind to the virus and prevent virus infection [5]. NA-targeted antibodies show a poorer neutralizing capacity, but they can slow the spread of the disease by blocking virus release from infected cells [6]. An influenza infection is primarily countered by antibodies to surface glycoproteins; however, the conserved proteins M and NP contained in the virion also elicit antibodies, but without neutralizing activity [7]. The cellular immune response promotes the apoptosis of the infected cells through virus-specific cytotoxic T-lymphocytes. These T-cells recognize antigenic epitopes of the viral internal proteins (matrix protein (M1) and the nucleoprotein (NP)) coupled with MHC molecules [8].

The influenza virus can escape recognition by the host immunity due to antigenic drift [9], which is the gradual accumulation of point mutations, eventually resulting in a virus with new antigenic properties. This is the reason why the antibodies created against the previous virus no longer recognize the newly emerged virus. Point mutations in the antigenic epitopes of internal proteins also contribute to the evasion of the cellular immune response [8]. The other type of change is called antigenic shift – the mechanism by which segments reassort to give rise to a virus with a pandemic phenotype [10]. The genome of the influenza virus consists of several segments, each of which behaves as an independent replication unit. This feature allows different influenza virus strains to combine and undergo genetic reassortment, which results in the emergence of reassortants. If two influenza A virus strains (avian and human) infect the same cell, packaging of segments from the two parental strains into one virion can occur, leading to the production of a hybrid progeny.

The role of other mechanisms in driving viral evolution, such as the emergence of defect particles [11] and intermolecular recombination, remains unclear. Although negative-strand RNA viruses with segmented genomes, to which the influenza virus belongs, rarely recombine, there is evidence that demonstrates the presence of cellular mRNA sequences in the HA gene. This propensity of the virus permits repeated infection cycles in trypsin-free cell cultures, which correlates with high virulence [12]. It is likely that similar mechanisms are behind the fast genetic changes seen in the repertoire of influenza A virus strains.

## THE VARIETY OF INFLUENZA A VIRUS STRAINS IN NATURE AND THEIR EVOLUTION

The influenza A virus strains found in animal and avian wildlife populations and recovered from humans exhibit a considerable degree of variation in their surface glycoproteins HA and NA. There are 18 known HA subtypes (H1–H18) and 11 known NA subtypes (N1–N11) [13]. Precursors to future pandemics could be viruses carrying the HA subtypes H1, H2, H3, H5, H6, H7, H9, H10, and NA subtypes N1, N2, N3, N8 that have been known to cause outbreaks or sporadic human infections. The most severe influenza pandemic ever recorded was the Spanish flu outbreak in 1918 that claimed from 50 to 100 million lives. This makes it extremely important to have models in place to predict such future disasters.

Seasonal epidemics are readily preventable with WHO recommended vaccines. But as a result of the fast evolution of a virus, the composition of such vaccines should be updated almost every year. Gaining insights into viral phylodynamics would play a crucial role in forecasting which viral subtypes are likely to affect the human population (epidemic or pandemic) and formulating a vaccine against the new strain.

Since an influenza virus evolution can be traced in real time, the field has seen an exciting flurry of methodological developments and experimental findings in the past decade.

## THEORETICAL MODELS TO PREDICT THE EVOLUTIONARY DYNAMICS OF THE INFLUENZA A VIRUS

Here, we will review the approaches that, in our opinion, are very promising for predicting the evolutionary dynamics of the influenza A virus. Such approaches involve the construction of phylogenetic trees based on the alignment of viral sequences and mathematical modeling (integro-differential equations, statistics, probability tests, simulation modeling) [14, 15].

Phylogenetic trees show the evolutionary relationship among different species or distant species sharing a common ancestor. The inference of such dendrograms includes the following steps: 1) a search for a cognate nucleotide and amino acid sequences; 2) multiple alignment; 3) construction of a phylogenetic tree using an algorithm of choice (for example, maximum likelihood, bootstrap analysis, matrix method, maximum parsimony); and finally 4) viewing and editing the tree structure. Currently, there are open access software and resources available online for a phylogenetic analysis of influenza A virus sequences [16].

One of the approaches mentioned above was used to examine the positive effect of a coordinated evolution on the influenza A virus fitness [17, 18]. The phenomenon when a mutation in one gene facilitates a mutation in another gene is called epistasis. The use of NA and HA amino acid sequences (H3N2 and H1N1 subtypes) retrieved from NCBI’s Influenza Virus Resource [19] to develop a statistical technique allows one to detect the potential pairs of sites involved in inter-gene epistasis. This approach uses the bootstrap algorithm. The approach is based on the identification of epistatic mutations in pairs of leading and trailing sites and the estimation of the distances between them in the tree. If the calculated distances are dramatically lower than the average distances, the mutations are considered epistatic according to the hypothesis that a mutation in one gene facilitates a mutation in another gene. However, these assumptions are not taken into account when it comes to the formulation of a vaccine, which could be very useful.

The other approach to influenza forecasting is the identification of clades (a population unit that is more than a single strain) in the phylogenic tree, which can show boom or bust dynamics of fitness in the subsequent season. A H3 subtype fitness model has been developed to predict influenza evolution trends on an annual basis [20]. Fitness outputs inform the choice of vaccines against seasonal influenza. The concentration (frequency) of the fitness strain is defined as the ratio of hosts infected with this strain to the whole population of hosts diagnosed with influenza. Depending on the season, the clade frequency is expressed as the sum of all frequency trajectories of seasonal strains from a given clade. The fitness (evolution rate) is a parameter that could increase or decrease the frequency of strains that descend from recent common ancestors next season [14]. A phylogenetic tree is built using maximum likelihood.

A predictive fitness model for influenza A based on the above-mentioned tools requires a database which contains the most up-to-date and comprehensive collection of the nucleotide sequences of seasonal influenza viruses.

For a phylogenetic tree to reflect the true phylogenetic relationships, the input data should be thoroughly evaluated and meet the stringent inclusion criteria (availability of full genome sequences of influenza A viruses, geographical mapping and so on) that contribute to a more accurate estimation of actual evolutionary relationships.

A good strategy for validating a phylogenetic tree and the inclusion criteria is to compare escape mutants, derived from a certain parental strain, and other cognate sequence clusters, with a tree rooted in a common ancestor. Escape mutants are viral mutants with the ability to escape neutralization by a monoclonal antibody. If escape mutants are represented in a dendrogram, they should cluster along with the parental strain. If the strains fall into different clusters, that could be explained either by an error in the data set of sequences or tree inference algorithms.

## EXPERIMENTAL MODELS TO PREDICT INFLUENZA A EVOLUTION

Like mathematical models, experimental models also utilize the nucleotide sequences of seasonal influenza A viruses deposited in databases. Importantly, experimental work generates new data sets containing the sequences of escape mutants. The common technique to experimentally produce HA escape mutants was reported as far back as 1980 [21]. Following the selection of escape mutants, the three-dimensional structures of a protein and the corresponding gene sequences are combined to map the epitopes (or single amino acid residues) recognized by the neutralizing antibodies. Escape mutant epitopes are spread non-randomly throughout the 3D structure (protrusions, loops, pockets).

Antigenic epitopes targeted by antibodies were first discovered in a 3D structure of the H3 hemagglutinin protein. For 20 years (from 1981 to 2001), it remained the only subtype whose 3D hemagglutinin protein structure was resolved by an X-ray analysis [22, 23]. Among the well-studied antibody interaction sites of escape mutants are such putative pandemic subtypes as H1 [24-26], H2 [26, 27], H3 [26, 28], H5 [26, 29-31] and H9 [26, 32]. There is scarce information on H7 subtypes [33], and no information on H6 and H10.

Due to fast evolutionary rates, the antibody interaction sites of the HA molecule of escape mutants are constantly evolving, generating newer viruses. This fact prompts research not only into poorly studied or completely uncharacterized subtypes (H6, H7 and H10), but also aims at further understanding the HA interaction characteristics of newly emerged viruses evolving from well-studied subtypes (H1, H2, H3, H5, H9). This thus becomes a top priority when a human pandemic caused by a new influenza subtype occurs.

The forecasting of a influenza A evolution builds upon the variation dynamics of both surface glycoproteins hemagglutinin and neuraminidase. Importantly, the coordinated evolution of the two proteins shapes the epidemiological profile of seasonal influenza strains. Our understanding of this relationship induced studies of hemagglutinin and neuraminidase proteins using escape mutants nearly at the same time.

## ADDITIONAL EXPERIMENTAL MODELS AIMED AT PREDICTING INFLUENZA A EVOLUTION

The ability to predict the subtype that will cause the next influenza is not limited to the identification of interaction sites on surface proteins (such as hemagglutinin and neuraminidase), which are responsible for antibody production.

It is important to monitor the wild-type strains of the influenza A virus reported in the past to identify the emergence of a virus produced under laboratory conditions. It has been demonstrated that not all escape mutations generated in the laboratory can occur in the influenza A virus under natural conditions. This discrepancy may be explained by the phenotypic effects triggered by mutations, necessitating a laboratory examination of such phenotypic characteristics of escape mutations as virulence, the ability to bind to cellular receptors (in avian and human hosts), replicative activity, virus yield at different temperatures, and finally resistance to environmental factors (temperature, pH).

For example, studies looking into the effect of amino acid substitutions in the HA protein on phenotypic change showed that the escape mutants of such putatively pandemic subtypes as H5 and H9 exhibit different variation patterns. H9 escape mutants do not vary much in phenotypic traits [34], whereas H5 escape mutants are very sensitive to single amino acid substitutions in the HA protein sequence [35, 36]. The RNA genome of the H9 subtype influenza virus shows lower evolutionary rates as compared to H5 subtypes in the wild. This fact is in agreement with experimental findings [34].

Overall, insights into the role of amino acid substitutions in escape mutant phenotypes will help guide our choice of experimentally produced clones with a fitness advantage and predict the epidemiological behavior of selected strains in the environmental context.

Not only mutations in surface glycoproteins, but also other capsid proteins could underlie the phenotypic variation in influenza virus A strains. Hence, to reliably confirm the association between the phenotype and a mutation in a protein (like hemagglutinin or other proteins), influenza viruses with the desired mutations should be prepared *in vitro *using reverse genetics and screened for phenotypic changes. Such an approach will thus support and narrow the diversity of predicted viruses.

Forecasting evolutionary trajectories towards pandemic H5 subtypes requires careful attention to hot spot mutations in the HA molecules that could contribute to high pathogenicity. The hot spots are:

• the receptor-binding site responsible for the attachment of the virus to the host cell surface; ;

• the sites involved in the binding to antibodies (antigenic epitopes);

• the glycosylation sites playing a role in the HA maturation process; and

• the proteolytic cleavage site of the hemagglutinin responsible for high pathogenicity.

This demonstrates the objective need for applying computer modeling and experimental findings to gain more in-depth knowledge of the evolutionary change in H5 influenza viruses in natural populations.

H5 subtype influenza viruses have been the focus of research since 1997, when this subtype was reported in humans [37]. The mortality rates caused by H5 subtypes of the influenza A virus hover around 53%, which is 5-fold higher than the notorious Spanish flu. There has been no report so far of human to human transmission for influenza viruses possessing H5 HA due to its high specificity to avian host cells [38]; however, upon conversion of H5 HA to an HA that could support efficient viral transmission in human populations, the pandemic would be the deadliest in human history.

The phylogenetic analysis of H5 sequences is hindered by incomplete sequence information in nucleotide databases. H5 sequences of escape mutants could enrich such databases, though it’s worth bearing in mind that experimentally produced escape mutants will serve as an approximation to a true evolutionary relationship among the identified viruses.

## WHAT PREVENTS AVIAN H5N1 FROM CROSSING THE SPECIES BARRIER TO INFECT HUMANS?

H5 viruses may acquire not only efficient transmission capability among humans, but also phenotypic fitness through mutations that may not take much time to occur.

**Fig. 1:**
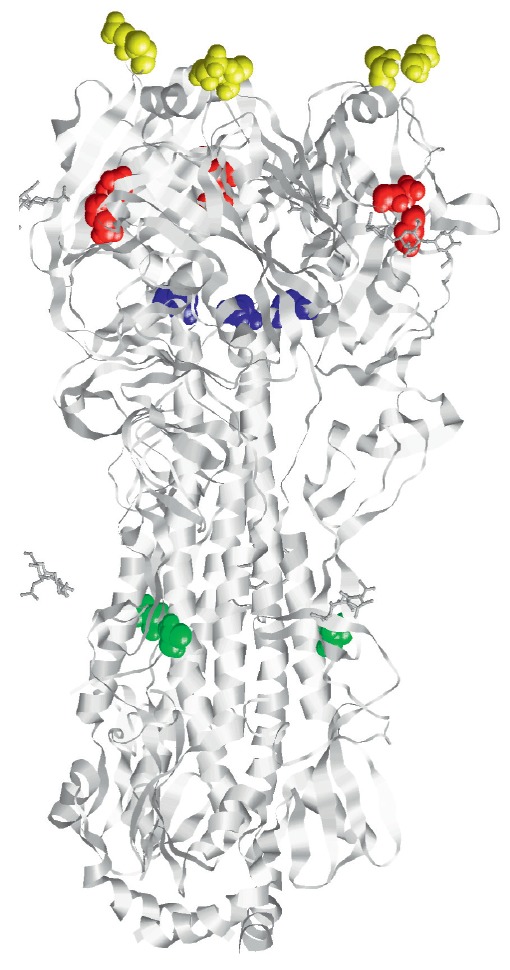
Positions of amino acids in the trimer H5 hemagglutinin protein, mutations at which contribute to the transmissibility of highly pathogenic H5N1 viruses among mammals [39, 40]

Experimental studies [39, 40] have shown that a few mutations in HA of the currently circulating H5N1 are sufficient for the virus to become a pandemic human influenza virus that spreads through respiratory droplets. These mutations (*Fig. 1*) are located at the receptor binding site (N224K, Q226L are in red), in the stalk region (T318I is in green) in the HA trimer-interface (H107Y is in blue), and at the glycosylation site (N158D, T160A are in yellow). Zhang et al. predicted amino acid substitutions in the HA protein that contribute to H5N1 transmissibility in mammals [41]. The positions at residues 186, 226, and 228 are located at the receptor binding site and at residue 160 at the glycosylation site. Two of these positions were predicted by computer modeling and further confirmed in field studies. Of note, the predicted positions reside in important regions of the HA molecule: the receptor binding site and the glycosylation site. More importantly, the position at residue 186 found in a laboratory-generated escape mutant is among those predicted computationally [36]. It was recently demonstrated that the HA molecule carries new (evolutionarily successful) positions, mutations at which confer fitness advantage and are coupled to changes toward a human-type receptor specificity of highly pathogenic H5N1 [42].

Overall, a comprehensive structural and functional evaluation of the receptor binding site, antigenic epitopes, the cleavage site, and the glycosylation site of various influenza A viruses would lay the groundwork for analyzing the evolutionary trajectories of circulating subtypes and offer new possibilities for predicting the natural emergence of new clones that are selected under laboratory conditions.

## ADDITIONAL PARAMETERS TO CONSIDER WHEN PREDICTING AVIAN INFLUENZA EVOLUTIONARY PATTERNS

**Fig. 2:**
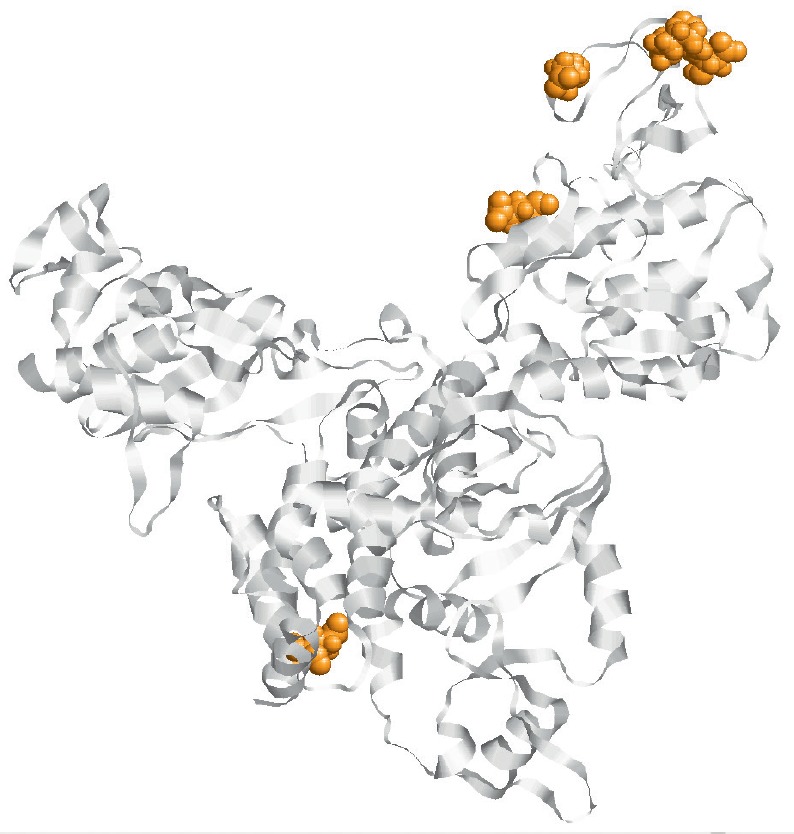
Positions of amino acids in the monomer PB2 protein, mutations at which contribute to the transmissibility of highly pathogenic H5N1 viruses among mammals [44]

The mammal-to-mammal transmissibility in highly pathogenic H5N1 is determined by not only HA changes, but also mutations in the PB2 polymerase subunit, in particular, the cap-dependent endonuclease responsible for the initiation of viral mRNA transcription and viral replicative ability [43]. It was recently shown that the genes of the polymerase subunit involved in the transmission to mammals contain mutations such as E192K, E627V, D701V, K702R on the PB2 subunit beside the substitution E627, (Fig. 2) and N105S on the PB1 subunit [44]. The key residues that contribute to a pandemic potential among mammals identified based on the phylogenetic analysis of the PB2 gene [45] include the positions 590, 627, and 701. The two residues at positions 627 and 701 predicted as precursors to a pandemic were in agreement with experimental findings [41].

Until recently, it was widely held that escape mutations cluster in regions of influenza surface proteins with high mutability. Recent findings have demonstrated that escape mutations may occur in conservative regions of internal proteins like the nucleoprotein (NP). NP was initially shown to be conservative. However, using a panel of monoclonal and polyclonal antibodies, it was found that the NP gene is subject to genetic change. Selection of influenza A NP escape mutants is not possible, since the NP protein does not elicit neutralizing antibodies. In this case, site-specific mutagenesis followed by ELISA evaluation of a protein produced in a prokaryotic vector could be used. All identified antigenically important amino acids in the NP protein were shown to be mutable and spread throughout the sequence as judged by a 3D structure [45]. Reports recently appeared on the location and structure of the compact antigenic site in the head domain of the influenza A virus NP protein [46].

This fact indicates that studies of evolutionary dynamics should look into mutations in both surface and internal proteins.

## CONCLUSIONS

The influenza A virus remains one of the most common and contagious human pathogens. It can cause epidemics and pandemics associated with high mortality and economic losses. These epidemiological traits are attributed to the high evolutionary rates and adaptability of A viruses to human hosts that possess a general innate immune system and varying levels of acquired immunity across individuals.

Since evolutionary dynamics can be tracked in real time, the influenza A research field has lately enjoyed a surge in experimental data (selection of escape mutants followed by phenotypic characterization, generation of viruses with the desired mutations using reverse genetics) and the development of novel techniques providing insights into the phylogenetic relationships of influenza strains, as well as mathematical (integro-differential equations, statistics, probability tests) and simulation modeling.

To ensure that the trees represent a true phylogenetic relationship among the viruses, input data should undergo quality control before being analyzed. The inclusion criteria are the availability of full genome sequences of influenza A viruses, geographical mapping and so on, which can make graphical representations more accurate. Escape mutants are a good option for validating tree-based models and, at the same time, verifying the selection criteria. In this case, all descending escape variants are compared against other viruses from different clades and the parental strain as an out group.

Relating changes in the amino acid sequences to phenotype allows one to limit the repertoire of selected escape mutants with a competitive advantage and predict their epidemiological behavior in nature. Phenotype changes result from not only mutations in the HA gene, but also other viral genes. Hence, a solid confir mation of the correlation between the HA genotype (or any other gene) and the phenotype should come from reverse genetics, whereby viruses with the desired mutations are constructed and examined for phenotypic characteristics in biological systems.

The phylogenetic analysis is impeded by incomplete data on the H5 sequences available in sequence repositories. To address this challenge, the H5 nucleotide sequences of escape mutants need to be submitted to such databases. However, it should be kept in mind that experimentally generated escape variants will be used as an approximation to a real evolutionary relationship among the viruses found in nature.

Both surface (HA and NA) and internal (NP, M1, M2, P) proteins are important when forecasting influenza A evolutionary patterns.

A combined use of state-of-the-art methods and the large body of experimental evidence should pave the way for more in-depth analyses of influenza A evolution.

## Abbreviations

HA: hemagglutinin of influenza virus
NA: neuraminidase of influenza virus
WHO: World Health Organization
MHC: major histocompatibility complex
H1–H18: subtypes of hemagglutinin of influenza A virus
N1–N11: subtypes of neuraminidase of influenza A virus
